# Association of the VEGFR2 single nucleotide polymorphism rs2305948 with glioma risk

**DOI:** 10.1097/MD.0000000000028454

**Published:** 2022-01-07

**Authors:** Shushu Sun, Xiaotian Li, Bingkun Qu, Kunming Xie, Jinlei Li, Junjie Miao

**Affiliations:** a Department of Infectious Diseases, Weifang People's Hospital, Weifang, China; b Department of Neurosurgery, Weifang People's Hospital, Weifang, China.

**Keywords:** glioma, rs2305948, single nucleotide polymorphism, vascular endothelial growth factor receptor 2

## Abstract

**Background:**

Many studies have reported a relationship between the vascular endothelial growth factor receptor 2 single nucleotide polymorphism (SNP) rs2305948 and glioma, but their conclusions have been controversial. A meta-analysis was performed to assess the association between rs2305948 and glioma susceptibility.

**Methods:**

Inclusion criteria and a strategy for screening of original literature were created. Eligible articles on the correlation between the SNP rs2305948 and glioma were identified in the PubMed, Embase, Web of Science, Cochrane Library, CNKI and Wanfang databases. After extracting the data, Stata 12. 0 software was used to perform statistical analysis under 5 genetic models and to calculate the combined odds ratio (OR) value and its 95% confidence interval (CI).

**Results:**

Four case-control studies including 1595 cases and 1657 controls were entered into the study. The overall analysis showed that no obvious association existed between rs2305948 and glioma risk (allele: OR = 1.20, 95% CI = 0.93–1.54, *P* = .162; dominant: OR = 1.17, 95% CI = 0.93–1.46, *P* = .174; recessive: OR = 1.72, 95% CI = 0.94–3.15, *P* = .076; heterozygous: OR = 1.11, 95% CI = 0.94–1.30, *P* = .226; homozygous: OR = 1.74, 95% CI = 0.92–3.29, *P* = .088). The subgroup analysis suggested that the SNP rs2305948 was related to glioma susceptibility under allele, dominant, recessive and homozygote models in the Asian population (allele: OR = 1.34, 95% CI = 1.16–1.55, *P* < .001; recessive: OR = 2.24, 95% CI = 1.49–3.36, *P* < .001; homozygous: OR = 2.32, 95% CI = 1.54–3.50, *P* < .001).

**Conclusion:**

The vascular endothelial growth factor receptor 2 rs2305948 gene polymorphism may be related to glioma susceptibility in the Asian population. However, the association is not clear in non-Asian populations, for which there has been less research.

## 1 Introduction

Glioma, a type of cancer that originates from glial cells, is the most common malignancy of the central nervous system.^[1]^ Statistics indicate that glioma accounts for more than 70% of intracranial malignancies.^[2,3]^ Glioblastoma is the most malignant type of glioma, and its incidence is approximately 3.2/100,000.^[4,5]^ Gliomas, particularly high-grade gliomas such as glioblastoma grow quickly, and the tumor cells easily invade the surrounding normal brain tissue because it is rich in blood vessels,^[6]^ thus, it is impossible to perform total surgical resection, and relapse commonly occurs after surgery.^[7]^ According to the latest statistics, even if the tumors are removed to the greatest extent possible and the patients receive temozolomide chemoradiotherapy in accordance with the treatment guidelines,^[8,9]^ the median survival is <2 years.^[10]^ The main reason for this poor survival is a lack of understanding of the nature of gliomas. Etiological research is the key to addressing the problems associated with these tumors. Most tumors result from the combined effects of environmental and genetic factors, and research has shown that genetic factors play important roles in carcinogenesis.^[11]^ Gliomas are vessel-rich tumors of the central nervous system. Vascular endothelial cell growth factor (VEGF) and VEGF receptor 2 (VEGFR2) are closely related to angiogenesis in glioma.^[12,13]^ Main functions of VEGFR2 include increasing the expression of VEGF and inducing tumor angiogenesis.^[14]^ In addition, VEGF plays a role in promoting vascular endothelial cell division and angiogenesis through VEGFR2 and is also involved in promoting the aggressive growth of tumors. Previous research has revealed that genetic mutations and polymorphisms are closely related to disease susceptibility and can lead to different responses to environmental factors and drugs.^[15–17]^ Thus far, many studies have reported an association between the VEGFR2 gene polymorphism rs2305948 and glioma risk, but the results of individual studies have been inconsistent. Therefore, we conducted this meta-analysis to evaluate the association of rs2305948 with glioma and to obtain a stronger conclusion.

## 2 Methods

### 2.1 Search strategy

A comprehensive search of studies on the association between the VEGFR2 gene polymorphism rs2305948 and glioma published before March 2020 was performed with the PubMed, Embase, Web of Science, Cochrane Library, CNKI, and Wanfang databases. The following search terms were used: (((((“Glioma”[Mesh]) OR ((((((((((((Gliomas[Title/Abstract]) OR (Glial Cell Tumors[Title/Abstract])) OR (Glial Cell Tumor)) OR (Tumor, Glial Cell[Title/Abstract])) OR (Tumors, Glial Cell[Title/Abstract])) OR (Mixed Glioma[Title/Abstract])) OR (Gliomas, Mixed[Title/Abstract])) OR (Malignant Gliomas[Title/Abstract])) OR (Gliomas, Malignant[Title/Abstract])) OR (Glioma, Malignant[Title/Abstract])) OR (Mixed Gliomas[Title/Abstract])) OR (Malignant Glioma[Title/Abstract])))) AND ((“Polymorphism, Genetic”[Mesh]) OR (((((((((Polymorphism∗[Title/Abstract])) OR (Variants[Title/Abstract])) OR (Variant[Title/Abstract])) OR (Mutation[Title/Abstract])) OR (Mutations[Title/Abstract])) OR (SNP[Title/Abstract])) OR (Single Nucleotide Polymorphism[Title/Abstract])))) AND ((((“Vascular Endothelial Growth Factor∗”[Mesh]) OR (VEGF∗[Title/Abstract]))) OR (rs2305948[Title/Abstract])). All analyses were based on previous published studies, thus no ethical approval and patient consent are required.

### 2.2 Inclusion and exclusion criteria

The inclusion criteria were as follows: the study assessed the association between VEGFR2 rs2305948 and glioma, the study provided genotype frequency distribution information, the genotype frequency of the control group in the study was in accordance with Hardy–Weinberg equilibrium (HWE, *P* < .01), and the study was a case-control study. No restrictions on ethnicity or geographic region were established.

The following types of articles were excluded: review articles, case reports, and animal experiment reports; articles with incomplete data; and replicate reports.

### 2.3 Data extraction

The data extracted from the enrolled studies included the following information: the first author, publication time, country, sample size, and genotype frequency. The literature quality evaluation and data extraction were completed by 2 researchers. We conducted quality assessment for the included studies with the Newcastle–Ottawa Scale.^[18]^ Disagreements were resolved via discussion with a third researcher.

### 2.4 Statistical analysis

Stata software (version 12.0, Stata Corporation, College Station, TX, USA) was used to conduct data analyses. The odds ratios (ORs) and their 95% confidence intervals (CIs) were determined to assess the relationship between the genetic polymorphism rs2305948 and glioma susceptibility. In the analysis, the capital letter “T” was defined as the mutant allele of rs2305948, while the capital letter “C” was defined as the wild-type allele. TT is a homozygote of a mutant allele, CT is a heterozygote, and CC is a homozygote of a wild-type allele. The genetic models used in this study were as follows: an allele model (T vs C), a dominant model (TT + CT vs CC), a recessive model (TT vs CT + CC), a heterozygote model (CT vs CC), and a homozygote model (TT vs CC). Heterogeneity among the included studies was assessed by Q test, and a *P* > .1 suggested that there was significant heterogeneity among the results of the studies. A subgroup analysis based on ethnicity was carried out to detect the source of heterogeneity. In addition, sensitivity analysis was performed by excluding individual studies to evaluate the stability of the results. Finally, Egger test and Begg test were applied to assess publication bias.

## 3 Results

### 3.1 Literature search results and study characteristics

**Figure 1:**
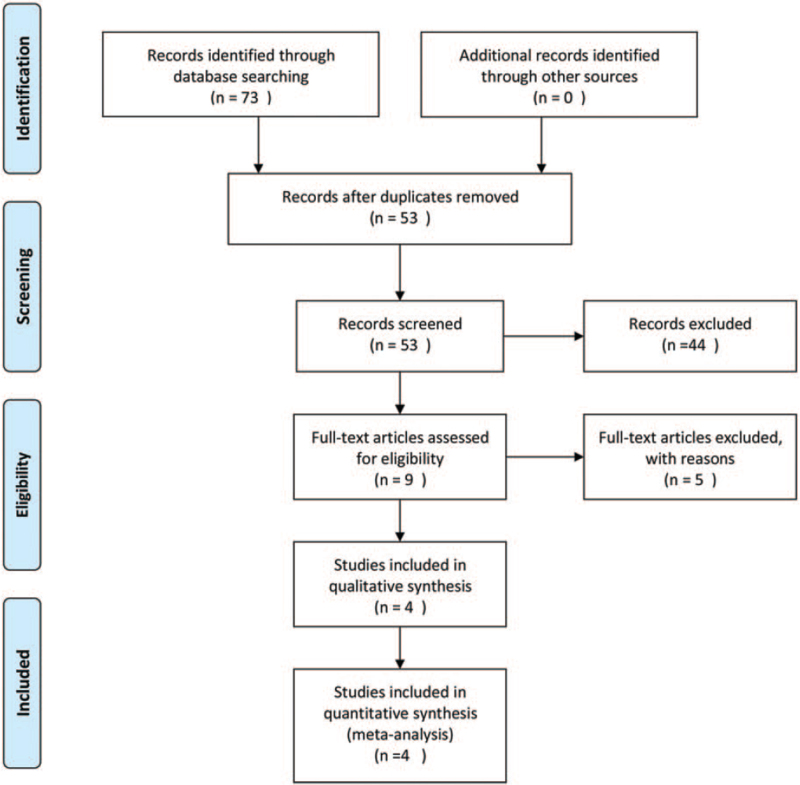
Flow diagram of the search strategy and literature selection.

A total of 73 relevant articles were included in the study after searching of the PubMed, Embase, Web of Science, Cochrane Library, CNKI, and Wanfang databases. Twenty duplicate articles were excluded, 44 articles were excluded after reading the title and abstract, and 5 articles were excluded after reading the full text. Ultimately, 4 articles were included in the meta-analysis. The characteristics of the included studies are shown in Table 1, and a flowchart of the literature search strategy is shown in Figure 1.

**Table 1:** Characteristics of included studies included in the meta-analysis.

						Case genotype			Control genotype				
Author	Year	Ethnicity	Study type	Genotyping method	Case/controls (n)	CC	CT	TT	CC	CT	TT	*P* ^∗^	Quality score
Vasconcelos	2019	Non-Asian	PB	RT-PCR	205/205	155	47	3	144	53	8	.274	7
Gao	2016	Asian	PB	PCR-RFLP	157/160	91	46	20	101	48	11	.122	8
Chen	2012	Asian	PB	MALDI-TOF mass spectrometry	756/815	558	171	27	629	173	13	.781	7
Zhang	2016	Asian	HB	MALDI-TOF mass spectrometry	477/477	332	117	28	364	101	12	.125	8

### 3.2 Statistical analysis results

**Figure 2:**
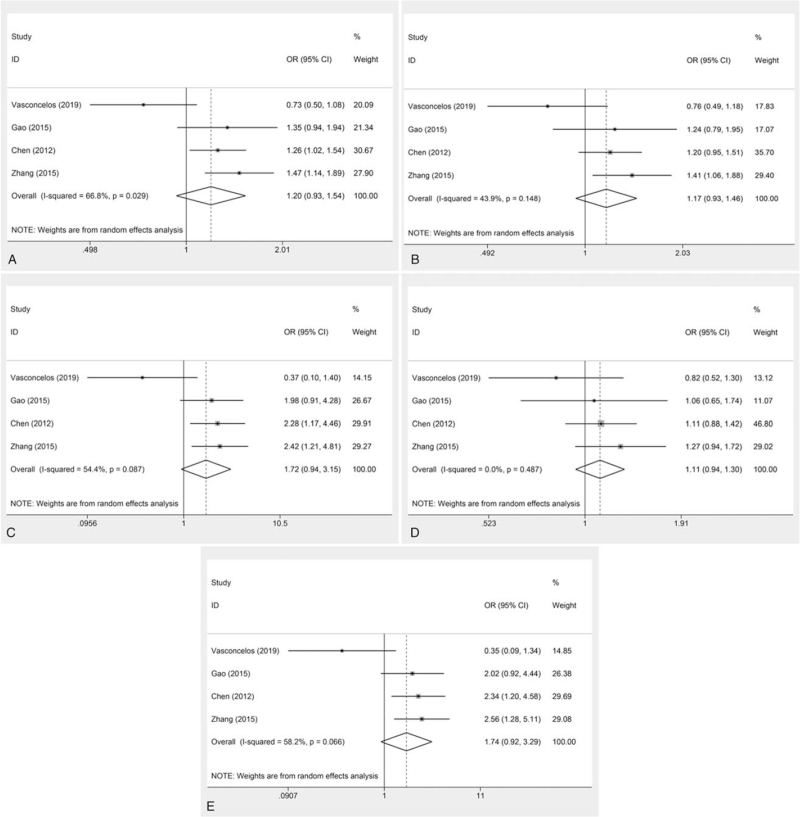
Forest plots of the meta-analysis for the association between the VEGFR2 rs2305948 polymorphism and glioma risk. A) allele model; B) dominant model; C) recessive model; D) heterozygote model; E) homozygote model. CI = confidence interval, OR = odds ratio.

**Figure 3:**
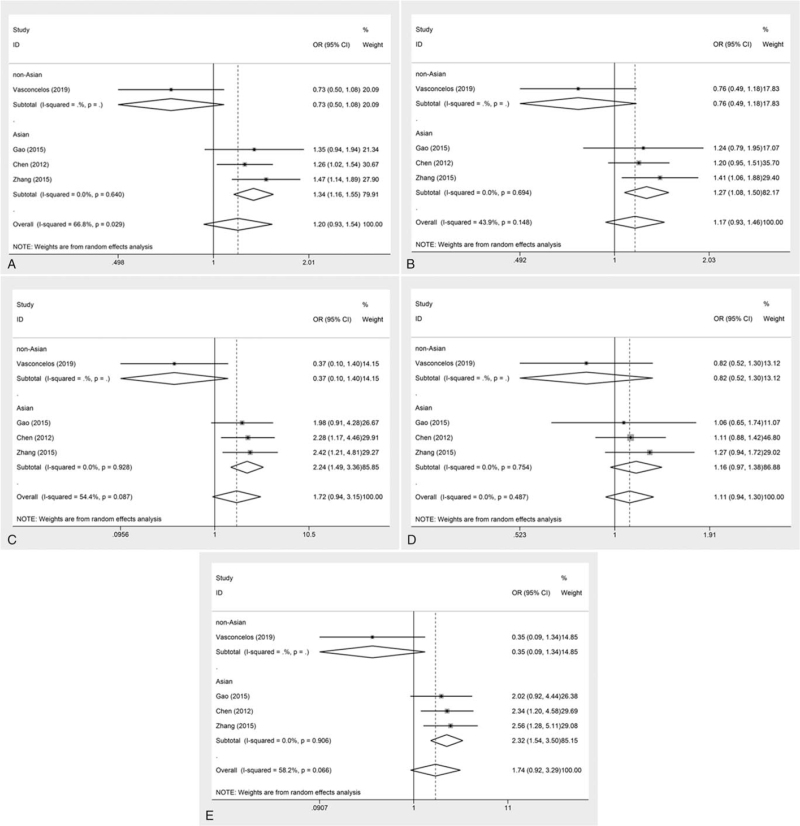
Forest plots of ethnicity subgroup analysis for the association between the VEGFR2 rs2305948 polymorphism and glioma risk. A) allele model; B) dominant model; C) recessive model; D) heterozygote model; E) homozygote model. CI = confidence interval, OR = odds ratio.

The overall analysis of 4 included studies showed that the association of the VEGFR2 gene polymorphism rs2305948 with susceptibility to glioma was not significant under each genetic model (allele: OR = 1.20, 95% CI = 0.93–1.54, *P* = .162; dominant: OR = 1.17, 95% CI = 0.93–1.46, *P* = .174; recessive: OR = 1.72, 95% CI = 0.94–3.15, *P* = .076; heterozygous: OR = 1.11, 95% CI = 0.94–1.30, *P* = .226; homozygous: OR = 1.74, 95% CI = 0.92–3.29, *P* = .088) (Fig. 2, Table 2). However, the ethnicity-based subgroup analysis showed that rs2305948 significantly increased the risk of glioma susceptibility in Asians (allele: OR = 1.34, 95% CI = 1.16–1.55, *P* < .001; recessive: OR = 2.24, 95% CI = 1.49–3.36, *P* < .001; homozygous: OR = 2.32, 95% CI = 1.54–3.50, *P* < .001) but was not significantly correlated with glioma risk in non-Asian populations (Fig. 3, Table 3).

**Table 2:** The association between VEGFR2 rs2305948 polymorphism and glioma risk.

	Heterogeneity-test				
Genetic model	*P* value	I^2^ (%)	Effect model	OR (95% CI)	*P* value
Allele model	.029	66.8	R	1.20 (0.93,1.54)	.162
Dominant model	.148	43.9	R	1.17 (0.93, 1.46)	.174
Recessive model	.087	54.4	R	1.72 (0.94, 3.15)	.076
Hemozygote model	.487	0	R	1.11 (0.94, 1.30)	.226
Homozygote model	.066	58.2	R	1.74 (0.92, 3.29)	.088

**Table 3:** Subgroup analysis of the association between VEGFR2 rs2305948 polymorphism and glioma risk.

		Heterogeneity-test				
Genetic model	Subgroup	*P* value	I^2^ (%)	Effect model	OR (95% CI)	*P* value
Allele model	Asian	.640	0.0	R	1.34 (1.16, 1.55)	<.001
Allele model	Non-Asian	–	–	R	0.73 (0.50, 1.08)	.117
Dominant model	Asian	.694	0.0	R	1.27 (1.08, 1.50)	.005
Dominant model	Non-Asian	–	–	R	0.76 (0.49, 1.18)	.222
Recessive model	Asian	.928	0.0	R	2.24 (1.49, 3.36)	<.001
Recessive model	Non-Asian	–	–	R	1.37 (0.10, 1.40)	.142
Hemozygote model	Asian	.754	0.0	R	1.16 (0.97, 1.38)	.105
Hemozygote model	Non-Asian	–	–	R	0.82 (0.52, 1.30)	.402
Homozygote model	Asian	.906	0.0	R	2.32 (1.54, 3.50)	<.001
Homozygote model	Non-Asian	–	–	R	0.35 (0.09, 1.34)	.518

### 3.3 Sensitivity analysis and publication bias

**Figure 4:**
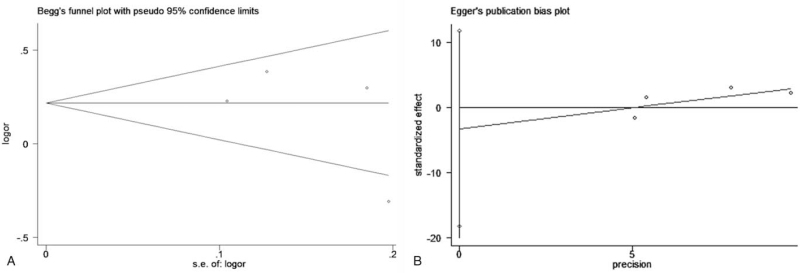
Funnel plot of Egger and Begg tests for publication bias Funnel plots of Egger and Begg tests for publication bias. A) Egger test; B) Begg test.

**Figure 5:**
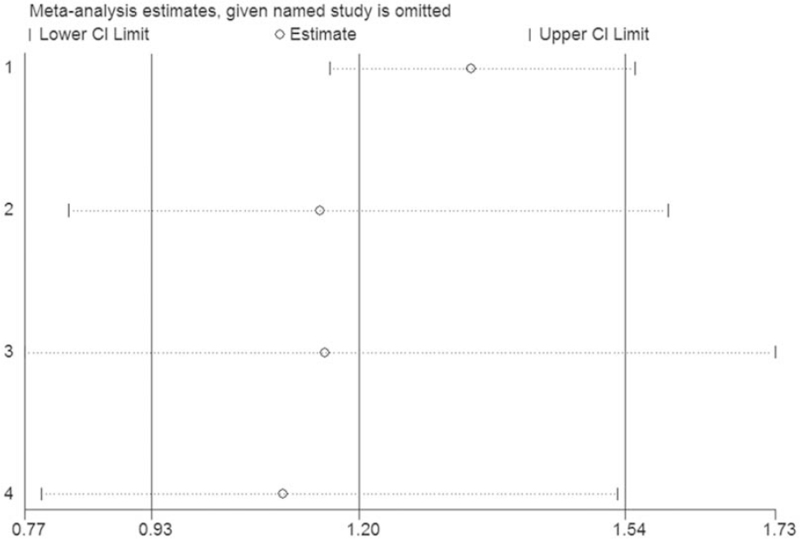
Sensitivity analysis of the association between the VEGFR2 rs2305948 polymorphism and glioma risk. CI = confidence interval.

Egger and Begg test were applied to detect publication bias, and the results revealed that there was no significant publication bias among the included studies (Fig. 4). The stability of the conclusions was assessed via sensitivity analysis, and the results suggested that the conclusions of the study are reliable (Fig. 5).

## 4 Discussion

Glioma is a common malignancy of the central nervous system. The pathogenesis of glioma results from a combination of genetic and environmental factors. Notably, the association of single nucleotide polymorphisms (SNPs) and tumor susceptibility has been a popular research topic in the field of cancer research. Importantly, genome-wide association study (GWAS) has become an effective method for detecting cancer susceptibility genes. Some genetic factors, such as pleckstrin homology-like domain family B member 1, telomerase reverse transcriptase, glutathione S-transferase P1, coiled-coil domain-containing 26, regulator of telomerase elongation helicase 1 and epidermal growth factor receptor genes, were confirmed to be related to glioma in genetic studies, including GWAS.^[19–23]^ However, the molecular mechanism underlying the occurrence and development of gliomas remains unclear, and to our knowledge, the VEGFR2 rs2305948 polymorphism has not been included in any GWAS or meta-analysis of glioma. We already know that the VEGFR family is composed of 3 transmembrane tyrosine kinase receptors, VEGFR1, VEGFR2, and VEGFR3, which play important roles in the process of blood vessel and lymphangiogenesis.^[24]^ VEGFR2 is generally considered one of the 3 receptors that plays a major role in mediating the VEGF-induced response VEGFR2 not only directly regulates tumor angiogenesis but also is overexpressed in various cancers, including breast, colon, ovarian, and lung cancer.^[25–28]^ Moreover, VEGFR2 is often amplified in many types of glioma, including low-grade glioma, glioblastoma, and recurrent glioma.^[29]^ Therefore, the abnormal expression of VEGFR2 may adversely affect the normal activities of nerve cells and eventually lead to the development of glioma. This effect may be achieved by increasing the expression of VEGFR2 via SNP rs2305948, but the detailed molecular mechanism still requires further study. Notably, rs2305948 is not only related to the incidence of the cancers mentioned above but also plays an important role in many other diseases. A previous study identified an close relationship between rs2305948 and coronary heart disease.^[30]^ In addition, a close relationship between rs2305948 and myocardial infarction in Caucasians has also been identified.^[31]^ Another study documented a close association between susceptibility to stroke and rs2305948.^[32]^ Many case-control studies on the relationship between the VEGFR2 polymorphism rs2305948 and glioma risk have been conducted, but the conclusions have not been consistent. Some studies have found that rs2305948 has no correlation with glioma susceptibility,^[33,34]^ while other studies have suggested that rs2305948 is closely associated with glioma susceptibility.^[35,36]^ Hence, we conducted this meta-analysis to analyze the association between rs2305948 and glioma susceptibility in order to draw reliable conclusions and provide up-to-date evidence for clinicians. The meta-analysis was conducted on 4 case-control studies including 1595 cases and 1657 controls. In our study, we applied 5 genetic models, which are the most commonly used genetic models in SNP research. The overall results suggested that no significant association exists between the rs2305948 polymorphism and susceptibility to glioma. A subgroup analysis based on ethnicity showed that rs2305948 increased glioma risk significantly in the Asian population under the allele, recessive and homozygous models. However, the association between rs2305948 and glioma risk was not clear in non-Asian populations because fewer studies on these populations were included; thus, there were smaller sample sizes for these populations than for the Asian population. In addition, we carried out a publication bias test because bias is an important factor influencing the credibility of conclusions,^[37]^ and the results showed that no significant publication bias existed. A sensitivity analysis demonstrated that the meta-analysis results were highly credible. Notably, heterogeneity is an important factor affecting the reliability of conclusions and should not be ignored.^[38]^ In the overall analysis, significant heterogeneity existed in the allele, recessive, and homozygous genetic models. To identify the sources of heterogeneity, we carried out a subgroup analysis based on ethnicity. We found that the heterogeneity was significantly reduced in the subgroups, suggesting that ethnicity may have been the cause of the heterogeneity.

This study had a few limitations. Few studies were included, especially studies on non-Asian populations, of which only 1 study selected local Brazilian residents was included. Most of the included studies were from Asian regions and were conducted on the Chinese Han population. In addition, due to a lack of data, subgroup analyses for age, sex, glioma grade, and other variables were not performed in the meta-analysis.

## 5 Conclusion

The results of the meta-analysis suggest that no significant correlation between the rs2305948 gene polymorphism and glioma susceptibility. However, subgroup analysis based on ethnicity demonstrated that rs2305948 may be associated with susceptibility to glioma specifically in Asian populations; the relationship among non-Asian populations was not clear due to a lack of research. Therefore, more case-control studies of reasonable design and with larger sample sizes, particularly GWAS of glioma, are needed to reveal the association between the VEGFR2 rs2305948 gene polymorphism and susceptibility to glioma.

## Author contributions

**Conceptualization:** Shushu Sun, Junjie Miao.

**Data curation:** Shushu Sun, Bingkun Qu.

**Formal analysis:** Shushu Sun.

**Investigation:** Xiaotian Li, Bingkun Qu.

**Methodology:** Xiaotian Li, Jinlei Li.

**Project administration:** Junjie Miao.

**Software:** Kunming Xie.

**Writing – original draft:** Shushu Sun.

**Writing – review & editing:** Junjie Miao.
